# Defense Mechanisms of *Xylopia aromatica* (Lam.) Mart. in the Dry Season in the Brazilian Savanna

**DOI:** 10.3390/life14111416

**Published:** 2024-11-02

**Authors:** Felipe Campos, Maria Vieira, Marília Sousa, Letícia Jorge, Gisela Ferreira, Marcia Marques, Carmen Boaro

**Affiliations:** 1 Biodiversity and Biostatistics Departament, Biosciences Institute, São Paulo State University (UNESP), Campus (Botucatu), P.O. Box 510, Botucatu 18618-970, SP, Brazil; maria.vieira@unesp.br (M.V.); marilia.caixetas@gmail.com (M.S.); leticia.g.jorge@unesp.br (L.J.); gisela.ferreira@unesp.br (G.F.); carmen.boaro@unesp.br (C.B.); 2 Agronomic Institute of Campinas (IAC), Plant Genetic Resources Center, Campinas 13075-630, SP, Brazil; marcia.marques@sp.gov.br

**Keywords:** Annonaceae, bicyclogermacrene, β-phellandrene, spathulenol, photosynthesis, antioxidant enzymes

## Abstract

Water availability and light during the dry and rainy seasons in the Cerrado may influence plants’ stomatal movement and the entry of CO_2_ for organic synthesis, which is the main electron drain. A lower stomatal conductance may contribute to the energy accumulated in the chloroplasts being directed towards the synthesis of compounds, which contributes to the activity of antioxidant enzymes to neutralize reactive oxygen species. *Xylopia aromatica* is a characteristic Cerrado species, and it is often recommended for recovering degraded areas. This study aimed to investigate the influence of the dry and rainy seasons on the metabolic adjustments of *Xylopia aromatica* in a portion of the Brazilian savanna in the state of São Paulo. In the rainy season, better photosynthetic performance led to greater investment in essential oil production. In the dry season, the plants may direct part of their reducing sugars to the syntheses of carotenoids and anthocyanins, which may help the antioxidant enzymes to neutralize reactive oxygen species. Carotenoids assist in the dissipation of photosystem energy, which has the potential to cause oxidative stress. During this season, lower stomatal conductance prevented excessive water loss. These results suggest the acclimatization of this species to the conditions of the Brazilian savanna.

## 1. Introduction

The Cerrado is a dry tropical forest in South America that covers a quarter of the national territory of Brazil and exhibits the greatest biodiversity in the world [1,2,3,4]. In recent decades, human actions, such as agricultural expansion and burning, have been the main causes of the degradation and fragmentation of the Brazilian savanna, resulting in the loss of around 50% of the original vegetation [1,5]. Therefore, studies involving local species are relevant to conserving the biodiversity of this Brazilian biome. It is particularly important to study the defense mechanisms used by species to survive in dry conditions. In this sense, *Xylopia aromatica* (Lam.) Mart. (Annonaceae) is a characteristic Brazilian savanna species, mainly recommended for the recovery of degraded areas of the Cerrado (SP) [6], Brazil (Resolução SMA nº 47/2003). *Xylopia aromatica* is also found in deciduous seasonal forests and the Amazonian savanna [6], being popularly known as pimenta-de-macaco, embira, pindaíba, and pimenta-de-bugre [7,8].

Climate change can cause variations in the dynamics of the Cerrado in both the dry and rainy seasons [1]. Thus, studying the interaction between plants and their changing environment contributes to understanding the impact on plant metabolism, helping to develop strategies for reforestation and preservation.

*Xylopia aromatica* seedlings introduced into a degraded area of the Cerrado as part of recovery efforts may present comparable nutritional status to those evaluated in preserved areas of the Cerrado. In addition, *Xylopia aromatica* has shown tolerance to high concentrations of leaf Fe and is therefore indicated for the recovery of areas contaminated with high levels of iron [9].

The metabolism of plant species in degraded environments can present, as a defense and acclimatization strategy, variations in both primary and specialized metabolism. Plants produce substances that belong to several chemical classes, which play important roles in their survival and adaptation to the ecosystem and contribute to their protection against abiotic and biotic stresses [10,11,12,13].

Phytochemical studies have shown that *Xylopia* contains several classes of substances that belong to specialized metabolic processes, including terpenes, amides, lignoids, alkaloids, and acetogenins [8,12,14,15]. Many of these substances have biological activity [16,17,18], including in humans, which may present variations in the different organs of the plant and with environmental conditions.

Among the terpenes, the monoterpenes α- and β-pinene and limonene, and the sesquiterpenes bicyclogermacrene and spathulenol, have been reported in the chemical profiles of essential oils extracted from the leaves, bark, flowers, fruits, and seeds of *Xylopia* plants in the Cerrado and Amazonian savanna [6,7,14,19,20,21,22].

Several studies have investigated the biological activity of *Xylopia aromatica* against biotic factors, such as fungi and bacteria. Nascimento et al. [21] evaluated the chemical profile and the antimicrobial and antifungal activity of the essential oils in the flowers and leaves of *Xylopia aromatica* collected in the Cerrado of Goiás, Brazil. The major compounds found in the leaves were spathulenol, khusinol, and bicyclogermacrene, and those found in the flowers included pentadecane-2-one, bicyclogermacrene, 7-epi-α-eudesmol, and khusinol. The essential oils of leaves and flowers showed lower minimum inhibitory concentrations (MIC) for *Streptococcus pyogenes* (200 and 100 μg mL^−1^, respectively). The essential oil in the leaves showed moderate inhibitory activity (500 μg mL^−1^) for *Candida albicans*. The essential oil obtained from the leaves of *Xylopia aromatica* of the Amazonian savanna, Amazonas, and Brazil showed a predominance of spathulenol, *trans*-pinocarveol, and dihydrocarveol, with strong activity against *Streptococcus sanguinis* [22]. Essential oil from *Xylopia aromatica* leaves collected in the Cerrado of Botucatu, SP, Brazil, contained α-pinene and β-pinene as major substances and showed antifungal activity against *Candida* and *Cryptococcus* species [23].

Another aspect of extreme importance, which has been less studied, is the effect the environment can have on the levels of enzymes that allow a plant to survive and regulate its primary and specialized metabolisms. Plants are sessile organisms subject to environmental conditions that can generate oxidative stress. This stress can be caused by variations in rainfall intensity, temperature, light, competition with other plants, pests, diseases, and herbivores. Under stress conditions, reactive oxygen species (ROS), molecules formed during normal metabolic functions in chloroplasts, mitochondria, and peroxisomes [24], are generated by the electron transport system, causing cellular damage or, depending on concentration, acting as signaling molecules that exert multiple defense responses. In these conditions, the plants depend on antioxidants for ROS neutralization and defense.

Antioxidant enzymes, including superoxide dismutase and peroxidases, can reverse oxidative stress [25]. These enzymes and the level of lipid peroxidation can be used [26] to monitor the damage caused by ROS and the malondialdehyde accumulation [27], acting as molecular markers to determine the stress level in plants.

The variation in water availability and light incidence in the Cerrado can influence the stomatal movement, as well as the entry of carbonic gas for organic synthesis, the main electron drain. A lower stomatal conductance may contribute to the accumulation of reducing agents in chloroplasts, which can be used in the synthesis of compounds that participate in specialized metabolism. This characterizes the non-enzymatic antioxidant system, which contributes to the activity of antioxidant enzymes to neutralize reactive oxygen species [28], such as mono- and sesquiterpenes. These substances are produced from the union of two or three isoprenes, respectively. The formation of isoprene requires a skeleton with five carbons, three ATP molecules, the reducing agent NAD(P)H^+^, and an electron donor source for the reduction of methylerythritol phosphate into isoprene. This isoprene route can also play a role in the dissipation of excess photosynthetic energy [28,29,30] and constitutes an important protection mechanism when the plant is subjected to a high light incidence. Under these conditions, drought or flooding stress may involve the emission of terpenes, which function as stress markers (as occurs in plant–plant and plant–insect interactions [31,32,33]) and act to stimulate a mechanism to overcome the stress factor.

No studies investigating the association between water availability and the defense mechanisms of the primary and specialized metabolisms of species native to the Cerrado have been found in the literature. This study aimed to investigate the influence of dry and rainy seasons on the metabolic adjustments in the photosynthetic and terpene profile of *Xylopia aromatica* in the fragment of Brazilian savanna in the state of São Paulo, Brazil.

## 2. Materials and Methods

### 2.1. Plant Material and Environmental Conditions

Twenty-eight individual *Xylopia aromatica* plants were evaluated. Fourteen were collected during the dry seasons, Dry1 (September 2016) and Dry2 (August 2017), and fourteen were collected during the rainy seasons, Rainy1 (February 2017) and Rainy2 (February 2018).

The evaluations were conducted on plants obtained from the Cerrado remnant at Rio Bonito, located in the municipality of Botucatu, state of São Paulo, Brazilian Southeastern region (geographical coordinates 22°42′09.4″ S, 48°20′64.1″ W and altitude of 514 m).

Reproductive materials from the species were collected, exsiccated, and deposited at the Herbarium Irina Delanova Gemtchujnicov (BOTU), located in the Biosciences Institute of Botucatu, UNESP, voucher nº 32478.

The chemical and physical characteristics of the collected soil samples were evaluated, and a pool consisting of 10 repetitions (20–40 cm) was made in the study area at each collection site. The chemical and physical characteristics of the soil showed differences in different seasons (Supplementary Table S1).

Measurements of precipitation (mm), relative humidity (%), and the average maximum and minimum atmospheric temperatures (°C) were obtained from the meteorological mini station (model PC400, Campbell Scientific, Shepshed, Loughborough, UK) located at the Unesp Biosciences Institute, Botucatu. The evaluations were carried out in the dry and rainy seasons, when the precipitation was lower and higher, respectively (Supplementary Figure S1).

### 2.2. Study Variables

The chlorophyll *a* fluorescence, gas exchange, leaf water potential, relative leaf water content, and yield and chemical profile of the essential oil were evaluated in 14 individuals in the Dry1 and Dry2 groups and another 14 individuals in the Rainy1 and Rainy2 groups.

Photosynthetic pigments, carbohydrates, antioxidant enzymes, and lipid peroxidation were evaluated in the Dry2 group and the Rainy2 group.

### 2.3. Chlorophyll a Fluorescence and Gas Exchange

The variables of fluorescence, potential quantum efficiency adapted to light (Fv′/Fm′), effective quantum efficiency (ɸPSII = ΔF/Fm′) of photosystem II (PSII), electron transport ratio (ETR = PPFD × ΔF/Fm′ × 0.5 × 0.84), heat dissipation in the antenna complex (*D* = 1 − Fv′/Fm′), and dissipation of excess energy from the PSII reaction center (*Ex* = Fv′/Fm′ × (1 − qP)) were determined [34,35]. The variables of gas exchange, CO_2_ assimilation rate (*Anet*, μmol CO_2_ m^−2^s^−1^), transpiration rate (*E*, mmol water vapor m^−2^s^−1^), stomatal conductance (*gs*, mmol m^−2^s^−1^), internal leaf CO_2_ concentration (*Ci*, μmol m^−2^ s^−1^ Pa^−1^), relative humidity (RH, %), instantaneous water-use efficiency (iWUE, µmol CO_2_ (mmol H_2_O)^−1^), and RuBisCO carboxylation efficiency (*Anet/Ci* (μmol m^−2^ s^−1^ Pa^−1^) [36] were determined using an open system of photosynthesis with a CO_2_ analyzer and infrared water vapor radiation (“Infra-Red Gas Analyzer—IRGA”, model GSF 3000, Walz, GmbH, Effeltrich, Germany) under a saturating irradiance of 1200 µmol m^−2^ s^−1^ (photosynthetic photon flux density (PPFD)), with a coupled modulated light fluorometer (LED-Array/PAM-Fluorometer 3055-FL, Walz, GmbH, Effeltrich, Germany). These measures were carried out in the period from 9:00 am to 11:00 am on a sunny day [37] with environmental CO_2_ equal to 415 ± 10 µmol m^−2^ s^−1^ and the following light intensities in the dry and rainy seasons: Dry1: 874.3; Dry2: 484.24; Rainy1: 873.21; and Rainy2: 900.07 µmol m^−2^ s^−1^ PPFD.

### 2.4. Water Potential and Relative Leaf Water Content

The water potential, represented in Mpa, of fully expanded leaves of the same individual was determined using a WP4-T water potential analyzer with a temperature controller (Decagon Devices, Pullman, WA, USA).

The relative leaf water content (RWC = (FM − DM)/(TM − DM) × 100) was determined using the weighing method for the fresh mass (FM), turgid mass (TM), and dry mass (DM) [38,39].

### 2.5. Yield and Chemical Profile of Essential Oil

The leaves of *Xylopia aromatica* were separated and dried at room temperature; essential oils were extracted by means of hydro-distillation in a Clevenger apparatus for 2 h. The essential oil yield was calculated as the mass of essential oil (g) per mass of dry plant material (g) and later expressed as a percentage.

The chemical composition was determined using a gas chromatograph coupled to a mass spectrometer (GC-MS—Shimadzu, QP-5000, Kyoto, Japan) operating at 70 eV. It was equipped with a DB-5 fused silica capillary column (30 m × 0.25 mm × 0.25 μm), with helium as the carrier gas (1.0 mL·min^−1^), an injector at 220 °C, a detector at 230 °C, a split of 1/20, and a temperature program of 60–240 °C, 3 °C min^−1^.

The substances were identified by comparing the obtained GC-MS with the system database, the linear retention index (LRI), and data from the literature [34]. The linear retention index (LRI) values of the substances were obtained by analyzing of the mixture of n-alkanes (C9–C24-99%, Sigma Aldrich, São Paulo, Brazil) under the same operating conditions as the samples, applying the Van Den Dool and Kratz [35] equation.

The quantification of the essential oil was performed using gas chromatography with a flame ionization detector (GC-FID), with the area normalization method and the operating conditions described above.

### 2.6. Photosynthetic Pigments, Carbohydrates, Antioxidant Enzymes, and Lipid Peroxidation

Determinations were performed on fully expanded leaves. Photosynthetic pigments, chlorophyll a and b, carotenoids, and anthocyanin were extracted and quantified according to the methodology proposed in [40].

The reducing sugars [38], total soluble sugars [39,41], and starch [42] were also quantified.

The total soluble protein content [43]; the activities of peroxidase (POD, EC 1.11.1.7) [44], superoxide dismutase (SOD, EC 1.15.1.1), and catalase enzymes (CAT, EC 1.11.1.6) [45]; and lipid peroxidation [46] were also determined.

### 2.7. Statistical Analysis

Variables were evaluated via analysis of variance (ANOVA), and the means were compared using the Tukey test at 5% probability in the SigmaPlot software, version 12.0 [47].

## 3. Results

### 3.1. Water Relations, Chlorophyll a Fluorescence, and Gas Exchange

**Figure 1:**
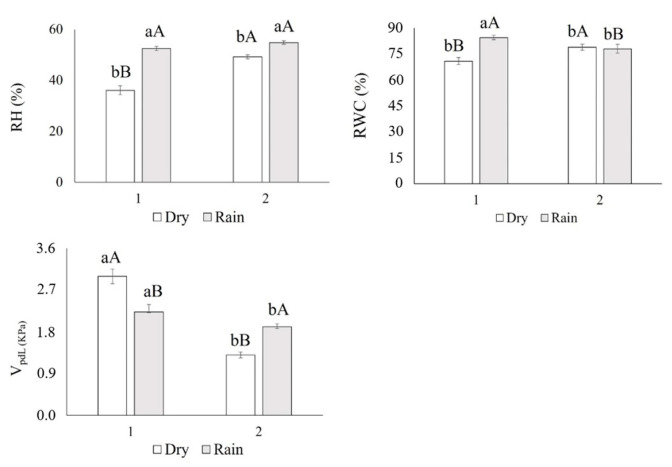
Relative humidity (RH, %) *p* ≤ 0.001, relative water content (RWC, %) *p* ≤ 0.001, and leaf vapor pressure deficit (VpdL, kPa) *p* ≤ 0.001 of *Xylopia aromatica* evaluated in the dry season (Dry1 and Dry2) and in the rainy season (Rainy1 and Rainy2) in the Brazilian savanna of Botucatu, SP, Brazil. Medium values. Capital letters test data through the years, lowercase letters test dry and rainy seasons. Means followed by the same letter did not differ from each other in the Tukey test at 5% probability.

**Figure 2:**
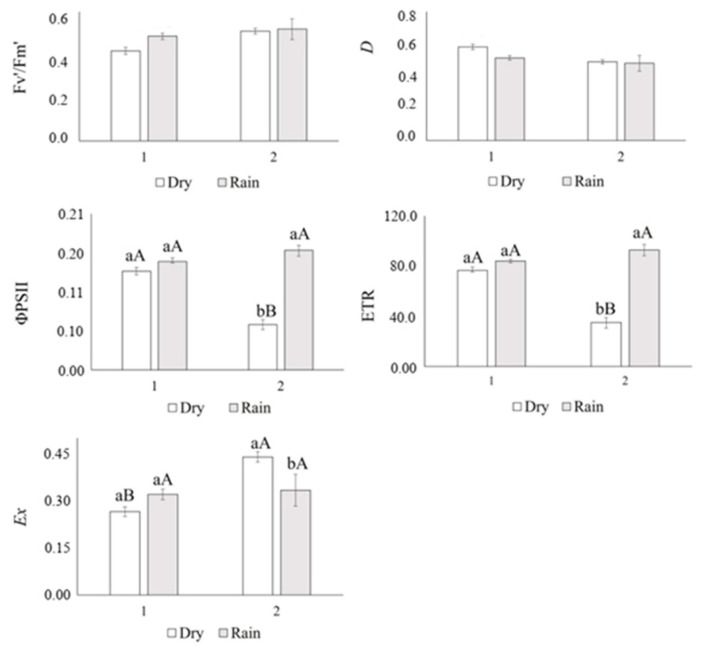
The efficiency of photosystem II (Fv′/Fm′) *p* < 0.288, the fraction of light absorbed by PSII antenna that is dissipated as heat (*D*) *p* < 0.288, effective quantum efficiency (ɸPSII) *p* ≤ 0.001, electron transport rate (ETR) *p* ≤ 0.001, and fraction of excitation energy not dissipated in the antenna that cannot be utilized for photochemistry (*Ex*) *p* < 0.008 of *Xylopia aromatica* evaluated in the dry season (Dry1 and Dry2) and in the rainy season (Rainy1 and Rainy2) in the Brazilian savanna of Botucatu, SP, Brazil. Medium values. Capital letters test data through the years, lowercase letters test dry and rainy seasons. Means followed by the same letter did not differ from each other in the Tukey test at 5% probability.

**Figure 3:**
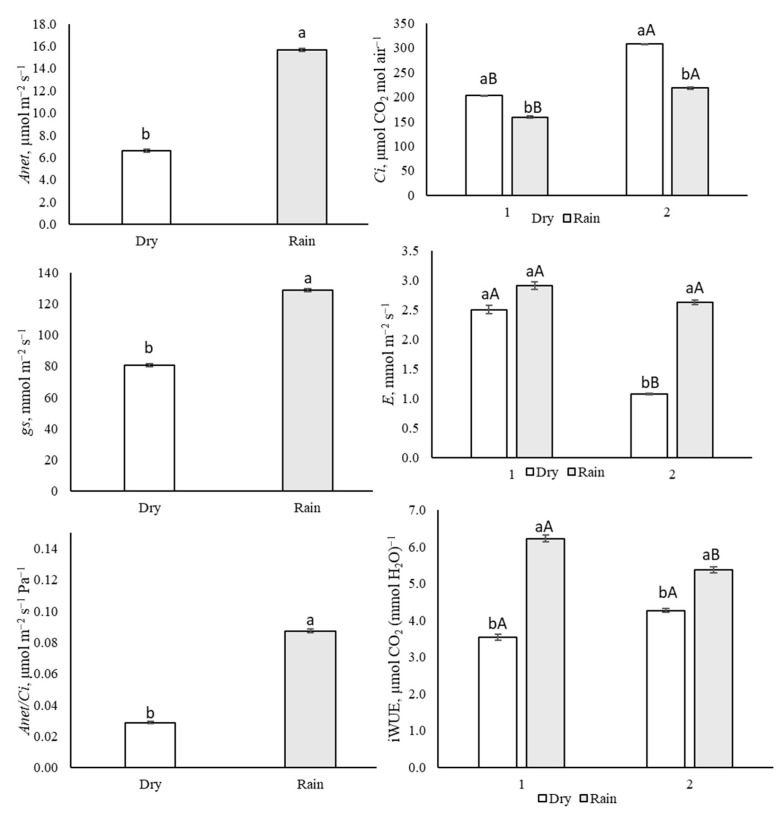
Leaf CO_2_ assimilation rate (*Anet*, μmol m^−2^ s^−1^) *p* ≤ 0.001, accumulation in the substomatal chamber (*Ci*, μmol CO_2_ mol Pa^−1^) *p* ≤ 0.001, stomatal conductance (*gs*, mmol m^−2^ s^−1^) *p* ≤ 0.001, transpiration rate (E, mmol m^−2^ s^−1^) *p* < 0.002, carboxylation efficiency (*Anet*/*Ci*, μmol m^−2^ s^−1^ Pa^−1^) *p* ≤ 0.001, and instantaneous water-use efficiency (iWUE, μmol CO_2_ (mmol H_2_O^−1^) *p* < 0.005 of *Xylopia aromatica* evaluated in the dry season (Dry1 and Dry2) and in the rainy season (Rainy1 and Rainy2) in the Brazilian savanna of Botucatu, SP, Brazil. Medium values. Capital letters test data through the years, lowercase letters test dry and rainy seasons. Means followed by the same letter did not differ from each other in the Tukey test at 5% probability.

The water potential (Ѱleaf) did not differ in the evaluated plants (*p* > 0.741). The relative humidity (RH%) and relative water content (RWC) were lower in the Dry1 season (Figure 1), while the vapor pressure deficit (VpdL) was greater (Figure 1). In general, in the rainy season (Rainy1 and Rainy2), the plants showed a higher electron transport ratio (ETR), effective quantum efficiency (ɸPSII), CO_2_ assimilation rate (*Anet*), stomatal conductance (*gs*), RuBisCO carboxylation efficiency (*Anet/Ci*), and instantaneous water-use efficiency (iWUE). The dissipation of excess energy from the PSII reaction center (*Ex*) was higher in the dry season (August 2017) (Figure 2 and Figure 3).

### 3.2. Yield and Chemical Profile of Essential Oil

**Figure 4:**
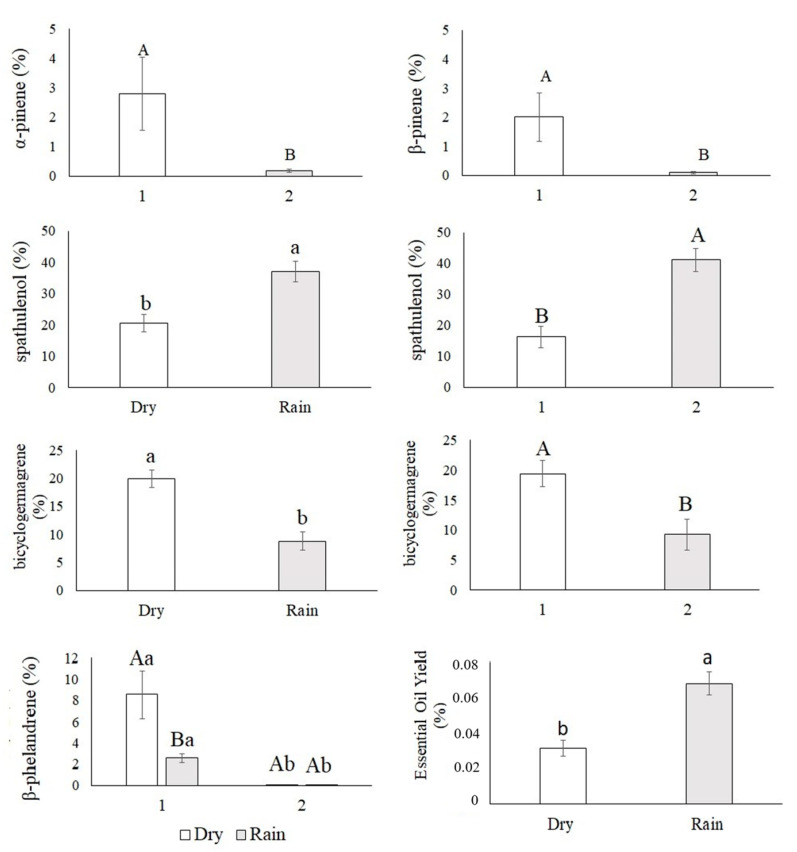
α-pinene (*p* < 0.043) and β-pinene (*p* < 0.030); spathulenol: season (*p* ≤ 0.001), year (*p* ≤ 0.001); bicyclogermacrene: season (*p* ≤ 0.001), year (*p* ≤ 0.001); β-phellandrene (*p* < 0.012), and essential oil yield of *Xylopia aromatica* evaluated in the dry season (Dry1 and Dry2) and in the rainy season (Rainy1 and Rainy2) in the Brazilian savanna of Botucatu, SP, Brazil. Medium values. Capital letters test data through the years, lowercase letters test dry and rainy seasons. Means followed by the same letter did not differ from each other in the Tukey test at 5% probability.

The yields of essential oil were 0.09% (Rainy1 season), 0.05% (Rainy2 season), 0.02% (Dry1 season), and 0.03% (Dry2 season), showing that the oil yield was greater in the rainy season (*p* ≤ 0.001) (Figure 4).

Although the chemical profiles of the essential oils of the 28 individuals of *Xylopia aromatica* were similar, there was variation in the relative proportion of their constituents in the four evaluated groups for the dry season (Dry1 and Dry2) and rainy season (Rainy1 and Rainy2) (Supplementary Table S2 and Figure 4).

The chemical classes identified in the essential oils were monoterpene hydrocarbons (α-pinene, sabinene, β-pinene, myrcene, α-phellandrene, o-cymene, limonene, β-phellandrene, and cis-β-ocimene) with average concentrations as follows: Dry1 season (15.56%), Rainy1 season (7.29%), Dry2 season (0.22%), and Rainy2 season (1.45%); sesquiterpene hydrocarbons (α-copaene, cis-caryophyllene, aromadendrene, germacrene D, bicyclogermacrene, and α-muurolene) with average concentrations as follows: Dry1 season (28.07%), Rainy1 season (18.46%), Dry2 season (19.87%), and Rainy2 season (3.60%); and oxygenated sesquiterpenes (spathulenol, caryophyllene oxide, and globulol) with average concentrations as follows: Dry1 season (15.84%), Rainy1 season (30.06%), Dry2 season (41.9%), and Rainy2 season (61.09%) (Supplementary Table S2).

The major constituents of the essential oils were β-phellandrene, α-pinene and β-pinene, bicyclogermacrene, spathulenol, and an unidentified substance (sub7). The Dry1 season showed a higher relative proportion of β-phellandrene (F = 7.157 *p* < 0.010). The substances α-pinene (F = 4.289 *p* < 0.043) and β-pinene (F = 4.999 *p* < 0.030) were higher in the first evaluation of both the Dry1 and Rainy1 seasons (Figure 4).

Bicyclogermacrene showed an increase in its relative percentage in the dry season and in evaluation 1, when the relative humidity was lower than in other evaluations (Supplementary Table S2 and Figure 4).

Spathulenol showed a higher relative proportion in the rainy season and assessment 2, a period of greater relative humidity (Supplementary Table S2 and Figure 4).

### 3.3. Photosynthetic Pigments, Carbohydrates, Antioxidant Enzymes, and Lipid Peroxidation

The chlorophyll a and b concentrations did not vary between seasons, while the carotenoid and anthocyanin concentrations were higher in the Dry2 season (Table 1).

The reducing sugars did not vary between seasons, and the concentrations of total sugars and starch were higher in the Dry2 season (Table 2).

The activities of the superoxide dismutase (SOD), peroxidase (POD), and catalase (CAT) enzymes and lipid peroxidation did not vary between seasons (Table 3).

## 4. Discussion

The plants evaluated in the Rainy1 season showed a higher photochemical efficiency, indicative of high production of reducing agents, despite the large amount of energy not dissipated or used in the photochemical phase (*Ex*), which did not promote stress.

The CO_2_ assimilation rate (Anet), stomatal conductance (gs), RuBisCO carboxylation efficiency (*Anet/Ci*), instantaneous water-use efficiency (iWUE), and relative water content (RWC) were higher in the Rainy1 season, when the plants presented lower vapor pressure deficits (VpdL). This condition allowed for greater stomatal apertures and increased the instantaneous water-use efficiency (iWUE) once the carbon input (*Anet*,) was greater than water output (E). These results agree with those recorded in [48].

In the Dry1 season, the lower relative humidity of the environment (RH%) and higher vapor pressure deficit (VpdL) resulted in partial closure of the stomata and lower stomatal conductance, a mechanism that contributed to the maintenance of water content and the regulation of leaf water potential (Ψleaf), which limited the entry of carbon and reduced its assimilation. These results agree with those obtained in [49] for Pinus palustres.

In the Dry2 season, plants showed the lowest effective quantum efficiency (ɸPSII) and electron transport rate (ETR), which may have resulted in lower production of reducing agents [50], limiting the synthesis of carbonic skeletons and causing the activation of alternative electron pathways, such as for the synthesis of carotenoids.

The greater amount of energy not dissipated or used in the photochemical phase (*Ex*) in the Dry2 season group should not be indicative of stress, since the concentrations of chlorophylls a and b did not change. The higher concentrations of carotenoids and anthocyanins must have helped the antioxidant enzymes in the neutralization of reactive oxygen species. Carotenoids in the xanthophyll pathway assist in the dissipation of photosystem II energy, which has the potential to cause oxidative stress [51].

These results agree with previous studies carried out in Eucalyptus globulus and Nicotiana attenuate [52,53]. In the present study, the increase in these pigments must have provided photoprotection and minimized the degradation of the membrane systems, since the amount of lipid peroxidation did not change.

Photoprotection can help prevent stress, which may explain the stability of antioxidant enzymes observed in the present study; this agrees with other results found in the literature [12,54].

Photoprotection can help prevent stress, which may explain the stability of the antioxidant enzymes observed in the present study; this agrees with other results found in the literature [12,54].

The finding of the highest concentration of starch in the dry season can be explained by the increased activity of the RuBisCO in the previous rainy season, storing surplus sugar for use in the unfavorable season, results that are confirmed in the literature [55]. This process probably maintains the concentration of reducing sugars in the dry and rainy seasons. These results suggest that in the rainy season, reducing sugars are derived from photosynthesis, while in the dry season, they come from the degradation of stored starch. These results suggest that the energy used in metabolism in the dry season may also be derived from starch degradation, as reported in [29].

In the dry season, *Xylopia aromatica* may direct part of its reducing sugars to the syntheses of carotenoids and anthocyanins, which would help explain the lower essential oil yield found in this study and in others [28,56,57].

*Xylopia aromatica* collected during the rainy season showed a higher electron transport rate, effective quantum efficiency, and stomatal conductance (*gs*), contributing to an increase in the rate of carbon assimilation and RuBisCO carboxylation efficiency (*Anet*/*Ci*). Therefore, the synthesis of a carbon skeleton for carbohydrate formation was possible, as revealed in a study carried out on rice [58]. It is suggested that, in the present study, carbohydrates formed in the rainy season provided carbon skeletons for essential oil synthesis. In this season the plants presented the highest effective quantum efficiency (ɸPSII), and instantaneous water-use efficiency (iWUE), which may have contributed to the higher leaf essential oil yield of *Xylopia aromatica* in the rainy season, as observed in [29].

In the Rainy1 season, a higher concentration of boron in the soil may have stimulated the synthesis of terpenes, evidenced by the greater production of essential oil. These results agree with those of [59], which suggested the participation of boron in moderate oxidative stress and essential oil production.

The relative proportions of the substances β-phellandrene, bicyclogermacrene, and spathulenol in the essential oil of *Xylopia aromatica* differed between sampling times, suggesting their influence on essential oil synthesis.

In the dry season, an unfavorable period for plant growth [60], there was a high proportion of β-phellandrene and bicyclogermacrene, suggesting the targeting of photosynthetic resources for the metabolism of these substances, which can help the activation of defense mechanisms and contribute to the acclimatization of the species to unfavorable conditions. These results verify previous findings [61,62].

*Solanum lycopersicum* presents β-phellandrene under osmotic stress conditions, and the substance is referred to as a stress indicator [31]. *Parthenium argentatum* has been shown to grow in conditions of moderate water deficit, displaying an increase in the relative percentage of β-phellandrene in its flower essential oil [32]. *Salvia dolomitica* cultivated in severe water deficit presented a two-fold increase in bicyclogermacrene content [33].

The high content of spathulenol in the rainy season is perhaps explained by the higher relative humidity, a favorable condition for the incidence and prevalence of phytopathogenic agents such as fungi [63]. Spathulenol presents antimicrobial activity [23,64], which can help control these pathogens.

## 5. Conclusions

*Xylopia aromatica* reveals photosynthetic and biochemical strategies that help it metabolically adjust to water availability, such as photoprotection mechanisms, stomatal control, starch accumulation, and terpene synthesis that contribute to the acclimatization of the species in the Brazilian savanna.

In the rainy season, better photosynthetic performance led to greater investment in essential oil production. In the dry season, the plants may direct part of their reducing sugars to the syntheses of carotenoids and anthocyanins, which must have helped the antioxidant enzymes neutralize reactive oxygen species. Carotenoids of the xanthophyll pathway assist in the dissipation of photosystem II energy, which can potentially cause oxidative stress. During this season, lower stomatal conductance prevented excessive water loss. These results suggest acclimatization of the species to the conditions of the Brazilian savanna.

## Figures and Tables

**Table 1:** Photosynthetic pigments, chlorophyll *a* (µmol g^−1^), chlorophyll *b* (µmol g^−1^), carotenoids (µmol g^−1^), and anthocyanins (µmol g^−1^) in *Xylopia aromatica* (Lam.) Mart. evaluated in the dry season and in the rainy season, in the Cerrado of Botucatu, SP, southeastern Brazil.

Seasons	Chlorophyll *a*	Chlorophyll *b*	Carotenoids	Anthocyanins
Dry	676.45 A ± 30.93	307.67 A ±18.89	392.67 ± 14.76 A	427.81 ± 25.27 A
Rainy	737.14 A ± 18.30	296.06 A ± 17.61	355.43 ± 12.51 B	319.82 ±19.17 B
*p*	0.09	0.616	0.046	0.01

Averages followed by the same letter do not differ from each other. Tukey (*p* < 0.05). Mean data (n = 14).

**Table 2:** Total soluble sugar (mg of total sugar g^−1^ of FW), reducing sugar (mg of reducing sugar g^−1^ of FW), and starch (mg starch g^−1^ of FW) in *Xylopia aromatica* (Lam.) Mart. evaluated in the dry and rainy seasons in the Cerrado of Botucatu, SP, southeastern Brazil.

Seasons	Total Soluble Sugar	Reducing Sugar	Starch
Dry	4198.50 ± 132.96 A	4.987 A ± 0.214	107.95 ± 3.519 A
Rainy	3530.82 ± 192.46 B	5.344 A ± 0.172	69.84 ± 6.699 B
*p*	0.005	0.269	≤0.001

Averages followed by the same letter do not differ from each other. Tukey (*p* < 0.05). Mean data (n = 14).

**Table 3:** Superoxide dismutase (SOD, U mg^−1^ protein), peroxidase (POD, µmol of purpurogallin min^−1^ mg^−1^ protein), catalase (CAT, μKat μg^−1^ protein), and lipid peroxidation (MDA, malondialdehyde nmol g^−1^ FW) in *Xylopia aromatica* evaluated in the dry season and the rainy season in the Brazilian savanna of Botucatu, SP, southeastern Brazil.

Seasons	SOD	POD	CAT	MDA
Dry	38.84 A ± 2.945	0.061 A ± 0.00014	0.15 A ± 0.00031	65.63A ± 2.0274
Rainy	39.73 A ± 2.752	0.061 A ± 0.00057	0.13 A ±0.00018	64.63 A ± 2.475
*p*	0.785	0.937	0.515	0.936

Averages followed by the same letter do not differ from each other. Tukey (*p* < 0.05). Mean data (n = 14).

## Data Availability

This published paper includes all data produced or analyzed during this project.

## Supplementary Materials

The following supporting information can be downloaded at: https://www.mdpi.com/article/10.3390/life14111416/s1, Figure S1: Chemical and physical characteristics of soil; Table S1: Chemical and physical characteristics of soil in the dry season—(september/2016—D1 and august/2017—D2) and in the rainy season—(february/2017—R1 and february/2018—R2), from the Rio Bonito region. Table S2: Relative percentage (%) of the essential oil substances of *Xylopia aromatica*.

## References

[1] Lopes dos Santos G., Pereira M.G., Delgado R.C., Magistrali I.C., Gomes da Silva C., Magno Moreira de Oliveira C., Pedro Bessa Larangeira J., Paula da Silva T. (2021). Degradation of the Brazilian Cerrado: Interactions with Human Disturbance and Environmental Variables. For. Ecol. Manag..

[2] Klink C.A., Machado R.B. (2005). Conservation of the Brazilian Cerrado. Conserv. Biol..

[3] Dias L.C.C., Moschini L.E., Trevisan D.P. (2017). A Influência das Atividades Antrópicas na Paisagem da Área de Proteção Ambiental Estadual do Rio Pandeiros, MG—Brasil. Fronteiras.

[4] Beuchle R., Grecchi R.C., Shimabukuro Y.E., Seliger R., Eva H.D., Sano E., Achard F. (2015). Land Cover Changes in the Brazilian Cerrado and Caatinga Biomes from 1990 to 2010 Based on a Systematic Remote Sensing Sampling Approach. Appl. Geogr..

[5] Fagundes N.C.A., Ferreira E.J. (2016). Veredas Da Região Sudeste: Peculiaridades Florísticas e Estruturais e Situação de Conservação. Neotrop. Biol. Conserv..

[6] Maas P., Lobão A., Rainer H. (2015). Annonaceae in Lista de Espécies da Flora do Brasil. http://floradobrasil2015.jbrj.gov.br/FB110557.

[7] Maia J.G.S., Andrade E.H.A., da Silva A.C.M., Oliveira J., Carreira L.M.M., Araújo J.S. (2005). Leaf Volatile Oils from Four Brazilian *Xylopia* Species. Flavour Fragr. J..

[8] Junqueira J.G.M., Do Nascimento M.N.G., Da Costa L.G., Romualdo L.L., De Aquino F.W.B., Abubakar M.N., Terezan A.P., Cunha G.O.S., Severino V.G.P. (2021). In Vivo and in Vitro Volatile Constituents of the Flowers of *Xylopia aromatica* by HS-SPME/GC-MS. J. Braz. Chem. Soc..

[9] Boni T.S., de Azevedo J.P., Oviedo Rodriguez A., Maltoni K.L. (2022). *Xylopia aromatica*: Crescimento Inicial e Status Nutricional de Mudas Em Solo Degradado Condicionado Com Resíduos. Res. Soc. Dev..

[10] Abbas F., Ke Y., Yu R., Yue Y., Amanullah S., Jahangir M.M., Fan Y. (2017). Volatile Terpenoids: Multiple Functions, Biosynthesis, Modulation and Manipulation by Genetic Engineering. Planta.

[11] Delazar A., Bahmani M., Shoar H.H., Tabatabaei-Raisi A., Asnaashari S., Nahar L., Sarker S.D. (2011). Effect of Altitude, Temperature and Soil on Essential Oil Production in Thymus Fedtschenkoi Flowers in Osko and Surrounding Areas in Iran. J. Essent. Oil-Bear. Plants.

[12] Spinelli F., Cellini A., Marchetti L., Mudigere K., Piovene C. (2011). Emission and Function of Volatile Organic Compounds in Response to Abiotic Stress. Abiotic Stress in Plants—Mechanisms and Adaptations.

[13] Soran M.L., Stan M., Niinemets Ü., Copolovici L. (2014). Influence of Microwave Frequency Electromagnetic Radiation on Terpene Emission and Content in Aromatic Plants. J. Plant Physiol..

[14] Fournier G., Hadjiakhoondi A., Charles B., Fourniat J., Leboeuf M., Cave A. (1994). Chemical and Biological Studies of *Xylopia aromatica* Stem Bark and Leaf Oils. Planta Med..

[15] Da Silva M.S., Tavares J.F., Queiroga K.F., De Fátima Agra M., Barbosa Filho J.M., Da Silva Almeida J.R.G., Da Silva S.A.S. (2009). Alcaloides e Outros Constituintes de *Xylopia langsdorffiana* (Annonaceae). Quim. Nova.

[16] Bakkali F., Averbeck S., Averbeck D., Idaomar M. (2008). Biological Effects of Essential Oils—A Review. Food Chem. Toxicol..

[17] Moura A.P.G., Beltrão D.M., Pita J.C.L.R., Xavier A.L., Brito M.T., de Sousa T.K.G., Batista L.M., de Carvalho J.E., Ruiz A.L.T.G., Della Torre A. (2016). Essential Oil from Fruit of *Xylopia langsdorffiana*: Antitumour Activity and Toxicity. Pharm. Biol..

[18] Aati H., El-Gamal A., Kayser O. (2019). Chemical Composition and Biological Activity of the Essential Oil from the Root of *Jatropha pelargoniifolia* Courb. Native to Saudi Arabia. Saudi Pharm. J..

[19] Oliveira V.B., Araújo R.L.B., Eidenberger T., Brandão M.G.L. (2018). Chemical Composition and Inhibitory Activities on Dipeptidyl Peptidase IV and Pancreatic Lipase of Two Underutilized Species from the Brazilian Savannah: *Oxalis cordata* A.St.-Hil. and *Xylopia aromatica* (Lam.) Mart. Food Res. Int..

[20] Andrade E.H.A., da Silva A.C.M., Carreira L.M.M., Oliveira J., Maia J.G.S. (2004). Essential Oil Composition from Leaf, Fruit and Flower of *Xylopia aromatica* (Lam.) Mart. J. Essent. Oil Bear. Plants.

[21] do Nascimento K.F., Moreira F.M.F., Alencar Santos J., Kassuya C.A.L., Croda J.H.R., Cardoso C.A.L., do Carmo Vieira M., Góis Ruiz A.L.T., Ann Foglio M., de Carvalho J.E. (2018). Antioxidant, Anti-Inflammatory, Antiproliferative and Antimycobacterial Activities of the Essential Oil of *Psidium guineense* Sw. and Spathulenol. J. Ethnopharmacol..

[22] Moreira Alcântara J., Mesquita J., De Lucena V.M., Facanali R., Ortiz M., Marques M., Da M., Lima P. (2017). Chemical Composition and Bactericidal Activity of the Essential Oils of Four Species of *Annonaceae* Growing in Brazilian Amazon. Nat. Prod. Commun..

[23] Vieira M.A.R., Jorge L.G., Marçon C., Campos F.G., Rozada A.M.F., de Freitas Gauze G., Seixas F.A.V., Marques M.O.M., Mendes R.P., Boaro C.S.F. (2023). Geographical Influences on the Chemical Composition and Antifungal Activity of *Xylopia aromatica* (Lam.) Mart. Leaf Essential Oil. S. Afr. J. Bot..

[24] Cao B.-L., Ma Q., Zhao Q., Wang L., Xu K. (2015). Effects of Silicon on Absorbed Light Allocation, Antioxidant Enzymes and Ultrastructure of Chloroplasts in Tomato Leaves under Simulated Drought Stress. Sci. Hortic..

[25] Spychalla J.P., Desborough S.L. (1990). Superoxide Dismutase, Catalase, and α-Tocopherol Content of Stored Potato Tubers. Plant Physiol..

[26] Erinle K.O., Jiang Z., Ma B., Li J., Chen Y., Ur-Rehman K., Shahla A., Zhang Y. (2016). Exogenous Calcium Induces Tolerance to Atrazine Stress in Pennisetum Seedlings and Promotes Photosynthetic Activity, Antioxidant Enzymes and PsbA Gene Transcripts. Ecotoxicol. Environ. Saf..

[27] Apel K., Hirt H. (2004). Reactive Oxygen Species: Metabolism, Oxidative Stress, and Signal Transduction. Annu. Rev. Plant Biol..

[28] Selmar D., Kleinwächter M. (2013). Stress Enhances the Synthesis of Secondary Plant Products: The Impact of Stress-Related over-Reduction on the Accumulation of Natural Products. Plant Cell Physiol..

[29] Loreto F., Schnitzler J.P. (2010). Abiotic Stresses and Induced BVOCs. Trends Plant Sci..

[30] Zhao L., Chang W., Xiao Y., Liu H., Liu P. (2013). Methylerythritol Phosphate Pathway of Isoprenoid Biosynthesis. Annu. Rev. Biochem..

[31] Tomescu D., Şumǎlan R., Copolovici L., Copolovici D. (2017). The Influence of Soil Salinity on Volatile Organic Compounds Emission and Photosynthetic Parameters of *Solanum lycopersicum* L. Varieties. Open Life Sci..

[32] Nik Z.B., Mirza M., Ghaffari M. (2008). Effect of Drought Stress on Growth and Essential Oil Contents in *Parthenium argentatum* Gray. J. Essent. Oil Bear. Plants.

[33] Caser M., Chitarra W., D’Angiolillo F., Perrone I., Demasi S., Lovisolo C., Pistelli L., Pistelli L., Scariot V. (2019). Drought Stress Adaptation Modulates Plant Secondary Metabolite Production in *Salvia dolomitica* Codd. Ind. Crops Prod..

[34] Adams R.P. (2017). Identification of Essential Oil Components by Gas Chromatography.

[35] van Den Dool H., Kratz P.D. (1963). A Generalization of the Retention Index System Including Linear Temperature Programmed Gas—Liquid Partition Chromatography. J. Chromatogr. A.

[36] Zhang S., Li Q., Ma K., Chen L. (2001). Temperature-Dependent Gas Exchange and Stomatal/Non-Stomatal Limitation to CO_2_ Assimilation of *Quercus liaotungensis* under Midday High Irradiance. Photosynthetica.

[37] Elsheery N.I., Cao K.-F. (2008). Gas Exchange, Chlorophyll Fluorescence, and Osmotic Adjustment in Two Mango Cultivars under Drought Stress. Acta Physiol. Plant.

[38] Miller G.L. (1959). Use of Dinitrosalicylic Acid Reagent for Determination of Reducing Sugar. Anal. Chem..

[39] Morris D.L. (1948). Quantitative Determination of Carbohydrates with Dreywood’s Anthrone Reagent. Science (1979).

[40] Sims D.A., Gamon J.A. (2002). Relationships between Leaf Pigment Content and Spectral Reflectance across a Wide Range of Species, Leaf Structures and Developmental Stages. Remote Sens. Environ..

[41] Yemm E.W., Willis A.J. (1954). The Estomation of Carboydrates in Plant Extracts by Anthrone. Biochem. J..

[42] Clegg K.M. (1956). The Application of the Anthrone Reagent to the Estimation of Starch in Cereals. J. Sci. Food Agric..

[43] Bradford M.M. (1976). A Rapid and Sensitive Method for the Quantitation of Microgram Quantities of Protein Utilizing the Principle of Protein-Dye Binding. Anal. Biochem..

[44] Teisseire H., Guy V. (2000). Copper-Induced Changes in Antioxidant Enzymes Activities in Fronds of Duckweed (*Lemna minor*). Plant Sci..

[45] Peixoto P.H.P., Pimenta D.S., Cambraia J. (2007). Alterações Morfológicas e Acúmulo de Compostos Fenólicos Em Plantas de Sorgo Sob Estresse de Alumínio. Bragantia.

[46] Heath R.L., Packer L. (1968). Photoperoxidation in Isolated Chloroplasts. Arch. Biochem. Biophys..

[47] Zar J.H. (2010). Biostatistical Analysis.

[48] Sage R.F., Way D.A., Kubien D.S. (2008). Rubisco, Rubisco Activase, and Global Climate Change. J. Exp. Bot..

[49] Addington R.N., Mitchell R.J., Oren R., Donovan L. (2004). a Stomatal Sensitivity to Vapor Pressure Deficit and Its Relationship to Hydraulic Conductance in *Pinus palustris*. Tree Physiol..

[50] Sharma A., Kumar V., Shahzad B., Ramakrishnan M., Singh Sidhu G.P., Bali A.S., Handa N., Kapoor D., Yadav P., Khanna K. (2020). Photosynthetic Response of Plants Under Different Abiotic Stresses: A Review. J. Plant Growth Regul..

[51] Baraldi R., Canaccini F., Cortes S., Magnani F., Rapparini F., Zamboni A., Raddi S. (2008). Role of Xanthophyll Cycle-Mediated Photoprotection in *Arbutus unedo* Plants Exposed to Water Stress during the Mediterranean Summer. Photosynthetica.

[52] Palmer-Young E.C., Veit D., Gershenzon J., Schuman M.C. (2015). The Sesquiterpenes(*E*)-β-Farnesene and (*E*)-α-Bergamotene Quench Ozone but Fail to Protect the Wild Tobacco *Nicotiana attenuata* from Ozone, UVB, and Drought Stresses. PLoS ONE.

[53] Machado F., Dias M.C., de Pinho P.G., Araújo A.M., Pinto D., Silva A., Correia C., Moutinho-Pereira J., Santos C. (2017). Photosynthetic Performance and Volatile Organic Compounds Profile in Eucalyptus Globulus after UVB Radiation. Environ. Exp. Bot..

[54] Gill S.S., Tuteja N. (2010). Reactive Oxygen Species and Antioxidant Machinery in Abiotic Stress Tolerance in Crop Plants. Plant Physiol. Biochem..

[55] Peng Y., Li C., Fritschi F.B. (2014). Diurnal Dynamics of Maize Leaf Photosynthesis and Carbohydrate Concentrations in Response to Differential N Availability. Environ. Exp. Bot..

[56] Arimura G.I., Matsui K., Takabayashi J. (2009). Chemical and Molecular Ecology of Herbivore-Induced Plant Volatiles: Proximate Factors and Their Ultimate Functions. Plant Cell Physiol..

[57] Chomel M., Guittonny-Larchevêque M., Fernandez C., Gallet C., DesRochers A., Paré D., Jackson B.G., Baldy V. (2016). Plant Secondary Metabolites: A Key Driver of Litter Decomposition and Soil Nutrient Cycling. J. Ecol..

[58] Seneweera S., Makino A., Hirotsu N., Norton R., Suzuki Y. (2011). New Insight into Photosynthetic Acclimation to Elevated CO_2_: The Role of Leaf Nitrogen and Ribulose-1,5-Bisphosphate Carboxylase/Oxygenase Content in Rice Leaves. Environ. Exp. Bot..

[59] Choudhary S., Zehra A., Naeem M., Khan M.M.A., Aftab T. (2020). Effects of Boron Toxicity on Growth, Oxidative Damage, Antioxidant Enzymes and Essential Oil Fingerprinting in Mentha Arvensis and *Cymbopogon flexuosus*. Chem. Biol. Technol. Agric..

[60] Llusia J., Roahtyn S., Yakir D., Rotenberg E., Seco R., Guenther A., Peñuelas J. (2016). Photosynthesis, Stomatal Conductance and Terpene Emission Response to Water Availability in Dry and Mesic Mediterranean Forests. Trees—Struct. Funct..

[61] Llusià J., Peñuelas J., Alessio G.A., Estiarte M. (2006). Seasonal Contrasting Changes of Foliar Concentrations of Terpenes and Other Volatile Organic Compound in Four Dominant Species of a Mediterranean Shrubland Submitted to a Field Experimental Drought and Warming. Physiol. Plant.

[62] Dudareva N., Klempien A., Muhlemann J.K., Kaplan I. (2013). Biosynthesis, Function and Metabolic Engineering of Plant Volatile Organic Compounds. New Phytol..

[63] Hussain M., Ismaili N.J. (2019). Phytopathogenic fungi associated with ripening fruit of date palm (*Phoenix dactylifera* L.) during rainy season in the university area of Khairpur, Sindh, Pakistan. Plant Prot..

[64] Tan N., Satana D., Sen B., Altan H.B., Demirci B., Uzun M. (2016). Antimycobacterial and Antifungal Activities of Selected Four Salvia Species. Rec. Nat. Prod..

