# Supplementary material for: Defense Mechanisms of Xylopia aromatica (Lam.) Mart. in the Dry Season in the Brazilian Savanna
Source: Life (Basel). 2024 Nov 2;14(11):1416. doi: 10.3390/life14111416 (PMC11595764; doi:10.3390/life14111416)
Supplement: Supplementary file 1 [file life-14-01416-s001.zip › Supplementary Table S1.pdf]

Supplementary Table S1. Chemical and physical characteristics of soil in the dry season - (september/2016 - D1 and august/2017 - D2) and in the rainy season - (february/2017 - R1 and february/2018 - R2), from the Rio Bonito region.

Season	pH	O. M.	P_{resina}	Al^{3+}	H+Al	K	Ca	Mg	SB	CEC	V%	B	Cu	Fe	Mn	Zn
	$CaCl_2$	g/dm³	mg/dm³	---------------mmol$_c$/dm³---------------								---------mg/dm³------------				
**D1**	3.9	14	2	11	46	0.3	2	1	3	48	6	0.48	0.3	83	0.7	0.2
**R1**	3.7	13	4	11	53	0.4	2	1	3	56	5	0.79	0.4	75	0.8	0.3
**D2**	3.9	14	5	10	43	0.6	2	1	3	46	7	0.35	0.3	73	1.3	0.4
**R2**	3.9	15	6	10	45	0.9	2	1	4	49	7	0.32	0.3	78	1.0	0.4

Organic matter (O.M.), cation exchange capacity (CEC), sum of bases (SB) and saturation by bases (V).
