# Supplementary material for: Defense Mechanisms of Xylopia aromatica (Lam.) Mart. in the Dry Season in the Brazilian Savanna
Source: Life (Basel). 2024 Nov 2;14(11):1416. doi: 10.3390/life14111416 (PMC11595764; doi:10.3390/life14111416)
Supplement: Supplementary file 1 [file life-14-01416-s001.zip › Supplementary Table S2.pdf]

Supplementary Table S2. Relative percentage (%) of the essential oil substances of *Xylopia aromatica* evaluated in dry season - (september/2016 - D1 and august/2017 - D2) and in the rainy season - (february/2017 - R1 and february/2018 - R2), in the Cerrado of Botucatu, SP, southeastern Brazil.

Substances	LIR_E	LIR_L	September/2016 - D1			February/2017 - R1			August/2017 - D2			February/2018 - R2		
			Min.	Max.	Mean	Min.	Max.	Mean	Min.	Max.	Mean	Min.	Max.	Mean
α-pinene	930	939	-	23.34	2.97	-	28.14	2.62	-	0.16	0.02	-	0.91	0.34
sabinene	971	976	-	1.73	0.37	-	0.29	0.16	-	-	-	-	-	-
β-pinene	976	980	-	17.18	2.30	-	18.04	1.70	-	-	-	-	0.63	0.18
mircene	958	991	-	2.87	0.52	-	0.68	0.06	-	-	-	-	0.23	0.03
α-phellandrene	1004	1002	-	2.55	0.40	-	0.96	0.12	-	-	-	-	-	-
o-cimene	1022	1022	-	2.59	0.21	-	-	-	-	0.28	0.03	-	1.76	0.48
limonene	1026	1028	-	-	-	-	-	-	-	0.93	0.08	-	1.23	0.38
sub1	1027	-	-	1.01	0.53	-	1.44	0.32	-	0.38	0.04	-	2.19	0.55
sub2	1028	-	-	5.52	1.90	-	3.89	0.49	-	-	-	-	-	-
β-phellandrene	1028	1029	1.67	33.26	8.56	-	6.15	2.56	-	-	-	-	-	-
cis-β-ocimene	1034	1040	-	0.72	0.22	-	0.22	0.05	-	-	-	-	-	-
α-copaene	1374	1376	-	0.56	0.09	-	0.7	0.24	-	0.69	0.17	-	0.95	0.17
cis-caryophyllene	1417	1418	0.45	1.40	0.86	-	0.94	0.45	-	1.28	0.56	-	0.93	0.12
sub3	1437	1439	-	3.80	1.83	-	1.95	0.90	-	1.99	1.18	-	0.35	0.03
aromadendrene	1437	1439	-	1.18	0.14	-	1.02	0.08	-	2.39	0.44	-	1.19	0.14
germacrene D	1479	1480	1.46	6.99	3.26	-	2.78	1.71	1.02	3.23	2.17	-	2.85	1.01
sub4	1481	1481	-	-	-	-	1.57	0.41	-	0.81	0.07	-	-	-
bicyclogermacrene	1494	1494	8.56	32.62	23.30	1.41	24.01	15.54	2.61	31.79	16.48	1.04	5.71	2.04
α-muurolene	1504	1500	-	1.75	0.42	-	0.84	0.44	-	0.56	0.05	-	0.79	0.12
sub5	1564	-	-	2.14	0.38	-	18.44	7.68	1.32	13.83	6.59	18.45	24.4	20.02
spathulenol	1574	1576	2.80	16.75	9.39	3.9	52.31	23.16	11.17	57.61	31.53	42.14	58.16	50.77
caryophyllene oxide	1579	1581	0.31	4.35	2.47	-	8.15	4.48	2.97	10.29	5.75	6.15	10.16	8.29
globulol	1578	1583	0.96	8.10	3.98	-	3.78	2.42	2.73	6.52	4.62	0.72	4.31	2.03
sub6	1740	-	-	4.90	1.66	-	1.2	0.68	-	4.69	2.07	-	1.38	0.74

sub7	**1762**	**-**	-	32.96	**19.16**	-	39.08	**18.03**	0.62	22.17	**9.33**	-	0.77	**0.10**
sub8	**1840**	**-**	-	6.14	**3.48**	-	18.41	**6.78**	-	24.31	**5.46**	-	-	**-**
sub9	**1853**	**-**	-	6.95	**1.70**	-	0.32	**0.03**	-	-	**-**	-	-	**-**
sub10	**1873**	**-**	-	2.23	**0.22**	-	-	**-**	-	11.13	**2.20**	-	-	**-**

LIR_E =Retention index experimental; LIR_L= Retention index literature (Adams, 2017); (-) ≤ 0.01
