# Supplementary figures and images for: Defense Mechanisms of Xylopia aromatica (Lam.) Mart. in the Dry Season in the Brazilian Savanna
Source: Life (Basel). 2024 Nov 2;14(11):1416. doi: 10.3390/life14111416 (PMC11595764; doi:10.3390/life14111416)

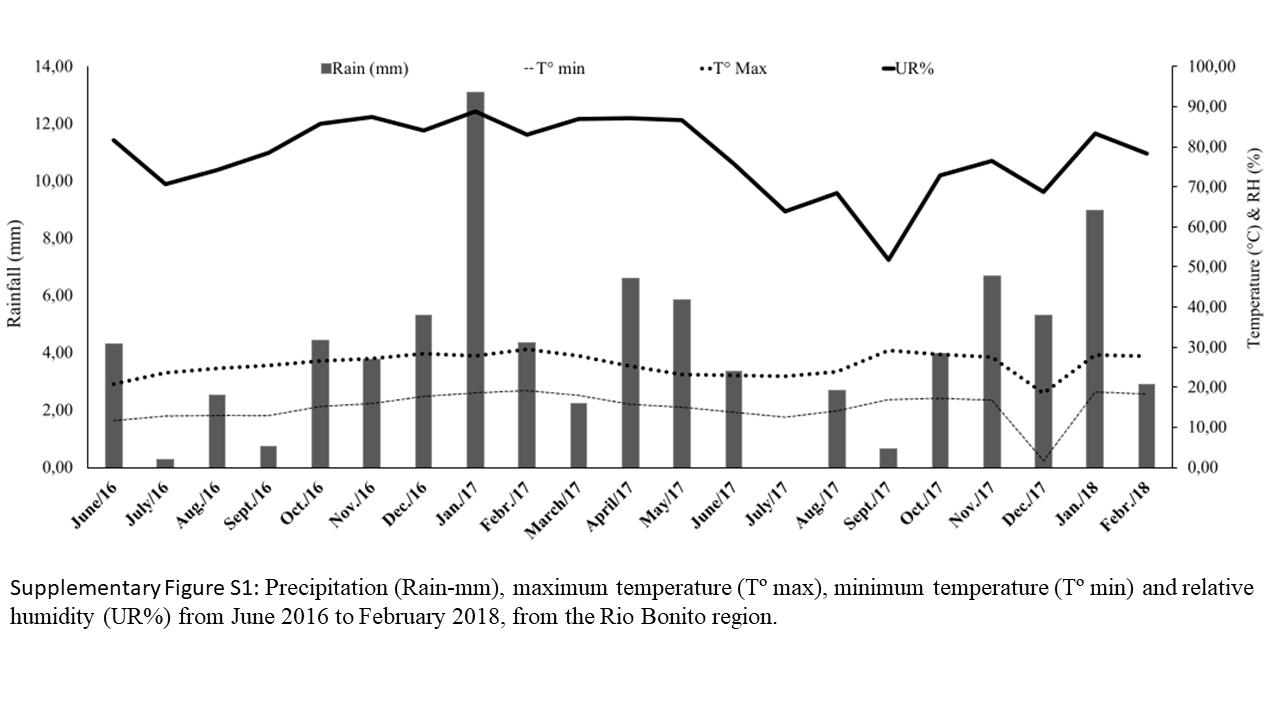

Supplement: Supplementary file 1 [file life-14-01416-s001.zip › Supplementary Figure S1.tif]
